# Biological characterization of *Aedes albopictus* (Diptera: Culicidae) in Argentina: implications for arbovirus transmission

**DOI:** 10.1038/s41598-018-23401-7

**Published:** 2018-03-22

**Authors:** Ailen Chuchuy, Marcela S. Rodriguero, Walter Ferrari, Alexander T. Ciota, Laura D. Kramer, María V. Micieli

**Affiliations:** 1 Centro de Estudios Parasitológicos y de Vectores (CEPAVE-CCT-La Plata-CONICET-UNLP), Boulevard 120 e/61y 62, 1900 La Plata, Argentina; 2 Departamento de Ecología, Genética y Evolución, Facultad de Ciencias Exactas y Naturales, Universidad de Buenos Aires, IEGEBA (CONICET-UBA), Intendente Güiraldes y Av. Costanera Norte s/n, 4to Piso, Pabellón II, Ciudad Universitaria, CI1428 EHA Ciudad Autónoma de Buenos Aires, Argentina; 3 The Arbovirus Laboratory, Wadsworth Center, New York State Department of Health, Slingerlands, NY United States of America; 4 Department of Biomedical Sciences, State University of New York at Albany School of Public Health, Albany, NY United States of America

## Abstract

*Aedes albopictus* (Diptera: Culicidae) is an invasive mosquito, native to Asia, that has expanded its range worldwide. It is considered to be a public health threat as it is a competent vector of viruses of medical importance, including dengue, chikungunya, and Zika. Despite its medical importance there is almost no information on biologically important traits of *Ae. albopictus* in Argentina. We studied life cycle traits, demographic parameters and analyzed the competence of this mosquito as a virus vector. In addition, we determined the prevalence of *Wolbachia* strains in *Ae. albopictus* as a first approach to investigate the potential role of this bacteria in modulating vector competence for arboviruses. We observed low hatch rates of eggs, which led to a negative growth rate. We found that *Ae. albopictus* individuals were infected with *Wolbachia* in the F1 but while standard superinfection with *w*AlbA and *w*AlbB types was found in 66.7% of the females, 16.7% of the females and 62.5% of the males were single-infected with the *w*AlbB strain. Finally, despite high levels of infection and dissemination, particularly for chikungunya virus, *Ae. albopictus* from subtropical Argentina were found to be relatively inefficient vectors for transmission of both chikungunya and dengue viruses.

## Introduction

*Aedes albopictus* (Skuse) (Diptera: Culicidae) is an invasive mosquito native to the forests of Asia that has expanded its range worldwide^1^. It is adapted to both tropical and temperate regions^2^ and to urban and peridomestic environments, where it feeds on humans and domestic animals and oviposits in a variety of natural and artificial containers^3^.

This mosquito is considered to be a public health threat because it is a competent vector of several viruses, including dengue,chikungunya, and Zika^4–10^. Although *Ae. albopictus* plays a relatively minor role compared to *Ae. aegypti* in DENV transmission, at least in part due to differences in host preferences and reduced vector competence^11^, recent outbreaks of chikungunya and dengue in Hawai, Mauritius, Gabon, Madagascar, and La Reunion^12^ and the first endogenous transmission of chikungunya in Europe^13^ by *Ae. albopictus* demonstrate the increasing public health impact of this mosquito species worldwide. Indeed, vector competence studies associated the emergence of chikungunya virus with a single mutation that enhances transmission efficiency by *Ae. albopictus*^14^.

*Ae. albopictus* was introduced in the mid-1980s into the United States and Brazil^15,16^. After this introduction, *Ae. aegypti* appeared to be outcompeted by *Ae. albopictus*^17^, but that is being reversed in some locations. Nonetheless, *Ae.albopictus* have been undergoing a rapid geographic expansion in the eastern U.S.^18^. Also, a geographic expansion of *Ae. albopictus* across Brazil^2^ to at least 20 of the 27 Brazilian states was recorded^19^. The Brazilian populations were shown to be related to the Southeast tropical Asian populations rather than the temperate Asian populations^20^. Biological traits, such as the winter diapause exhibited by the USA populations were not observed in the Brazilian populations^3^. In Argentina, *Ae. albopictus* was detected for the first time in 1998 in two localities in Misiones (North-eastern Argentina), close to the Brazilian border^21,22^, and 2002–2004 period in two other locations in Misiones^23^. To our knowledge, there is no other record of *Ae. albopictus* in Argentina and despite the risk it poses, there is almost no information on biological traits, such as, life cycle trait and demographic parameters.

This species is naturally super-infected with two *Wolbachia* strains, *w*AlbA and *w*AlbB^24^, which induce cytoplasmic incompatibility (CI), i.e. embryonic lethality in crosses between uninfected females and infected males (unidirectional CI) or between males and females infected with different strains (bidirectional CI)^25^. The lack of genetic variation in both *w*AlbA and *w*AlbB suggests that this reproductive parasite has recently invaded and spread throughout populations of this mosquito^26^. both strains have been shown to increase female fecundity^27,28^ and male mortality^29^, although the impact of the super-infection on the *Ae*. *albopictus* life-history traits would be better depicted as dependent of both sex and competition^30^. These *Wolbachia* strains have also been shown to induce resistance to viruses^31^, although at a lower level than mosquito-transinfected strains^32^. In order to determine the potential importance of *Ae*. *albopictus* in dengue (DENV) and chikungunya (CHIKV) transmission in Argentina, we studied biological features including life cycle traits, demographic parameters and vector competence. In addition, we determined the prevalence of *Wolbachia* strains in *A*e*. albopictus* as a first approach to investigate the potential role of this bacteria in modulating vector competence for arbovirus.

## Results

### Survey

**Figure 1:**
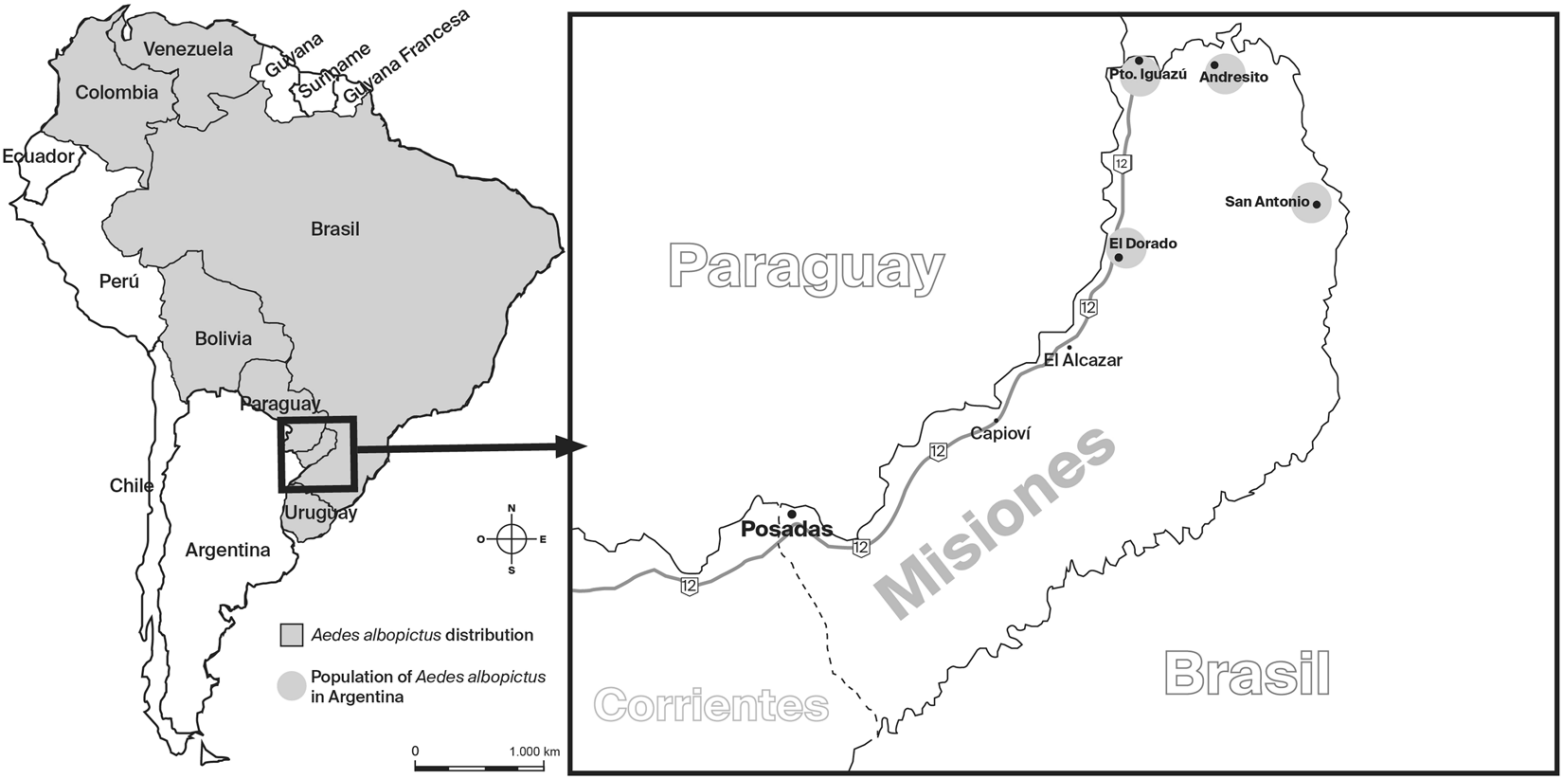
Distribution the *Ae. albopictus* populations in Argentina and South America including the field collection sites in Misiones province, Argentina. The collection sites are shown along the grey line (route 12) connecting Puerto Iguazú with Posadas in Misiones province. The map was created using Adobe InDesign CS6 sofware.

During the survey that took place along Route 12, that connects Puerto Iguazú with Posadas (Misiones, Argentina), immature stages of *Ae. albopictus* were found only in Iguazú National Park (Fig. 1).The relative abundance was low (90 larvae) compared with other mosquito species (*Culex* sp., 665 larvae and *Aedes aegypti*, 257 larvae) collected from three artificial containers.

### Laboratory life cycle traits and demographic parameters

A total of 49 adults were obtained (29 males and 20 females) from the 50 F1 larval specimens obtained in the field. Thus, the survival rate from first instar larvae to pupae was 98%. The sex ratio was 0.68 [♀/♂]. Fecundity was 30 eggs per fed female after the first blood feeding and 82 after the second feed. 10% of eggs laid hatched. The average longevity for adults was 16 days for females and 10 days for males. The demographic parameters were as follows: Net Reproductive Rate (R_0_) was 0.512, Cohort Generation Time (Tg) was 33 days and the Intrinsic Rate of Population Growth (r) was −0.0197.

### Wolbachia infection status in adult mosquitoes

Two out of 20 females of *Ae. albopictus* were discarded because DNA extraction failed. Therefore, a total of 18 females were analyzed. Overall, 83.3% of females were *Wolbachia* positive. Of these, 66.7% had double infection with the strains *w*AlbA and *w*AlbB, while 16.7% were infected only with *w*AlbB. From the eight males assayed, six tested positive for *Wolbachia*, indicating a 75% prevalence. We found that 62.5% had a single infection with the *w*AlbB strain, while 12.5% had a double infection.

Comparison of the sequences obtained for each locus of the full MLST (by alphabetical order) revealed the following allelic profile for *w*AlbB: (229-27-210-242-166). Also, sequencing of the *wsp* gene, which provides complementary information to the standard MLST system, indicated the following genotype for the four hypervariable regions of the gene (HVR1-4): 10-82-10-84. This information is reported for the first time for the *w*AlbB strain.

**Figure 2:**
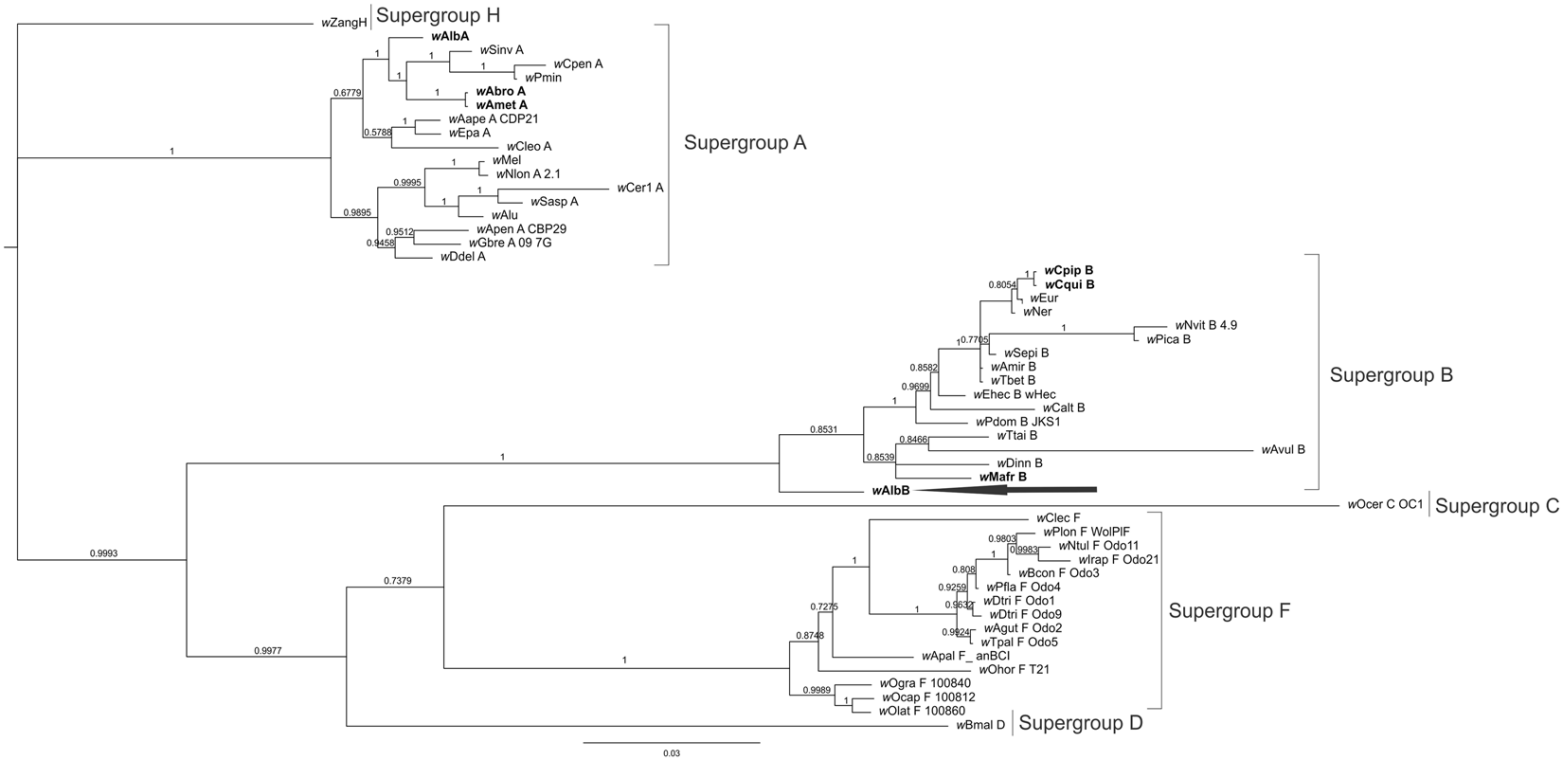
Phylogeny of *w*AlbB strain based on *Wolbachia* MLST genes.Tip labels include 52 *Wolbachia* strains from PubMLST (http://pubmlst/wolbachia) and strains from this study. Black arrows indicate positions of *w*AlbB strain. The black letters indicate *Wolbachia* strains corresponding to mosquitoes. Posterior probabilities are indicated by the number at the node. The tree was constructed using the Bayesian MrBayes sofware.

As expected, *w*AlbB strain grouped with other strains from the B supergroup (Fig. 2). As it was seen before for other mosquitoes, this strain is not closely related to other Culicidae strains from the same supergroup.

### Vector competence assay

The percent of *Ae. albopictus* females that became infected with DENV2 after feeding on infectious blood containing 4.5 × 10^6^ PFU/ml was 52.5% (N = 120 fed females). Dissemination was detected in 36.5% (N = 63) of infected females. Dissemination of virus varied from 0% (0/16) on day 5 post infection, 38.5% (5/13) on day 10, 41.1% (7/17) on day 14 and 64.7% (11/17) on day 21. Virus was not detected in the salivary secretions until day 21 with only one positive (4.3%, N = 23) (Table 1).

**Table 1:** Vector competence of DENV and CHIKV in *Ae. albopictus* population from Iguazú National Park, Misiones.

Virus	Transmission day	Blood meal titer	No. Fed mosq.	No. Infected/No. Fed mosq. (%)	No. Disseminated/No. Infected (%)	No. Transmitting/No. Disseminated (%)	No. Transmitting/No. Fed mosq.(%)
DENV 306	5		30	16/30 (53.3)	0/16 (0)	0/0 (0)	
	10	4.5 × 10^6^	30	13/30 (43.3)	5/13 (38.5)	0/5 (0)	
	14		30	17/30 (56.6)	7/17 (41.1)	0/7 (0)	
	21		30	17/30 (56.6)	11/17 (64.7)	1/11 (9)	
			120	63/120 (52.5)***	23/63 (36.5)**	1/23 (4.3)	1/120 (0,8)*
CHIKV 91077	2		30	18/30 (60)	0/18 (0)	0/0 (0)	
	5		30	22/30 (73.3)	9/22 (40.9)	0/9 (0)	
	10	1.7 × 10^7^	30	22/30 (73.3)	16/22 (72.7)	1/16 (6.2)	
	14		30	23/30 (76.6)	21/23 (91.3)	1/21 (3.8)	
	21		26	17/26 (65.3)	13/17 (76.4)	2/13 (15.3)	
			146	102/146 (69.8)***	59/102 (57.8)**	4/59 (6.7)	4/146 (2.8)*

Statistically significant differences in infection and dissemination between DENV and CHKV viruses in *Ae. albopictus* are shown by asterisk (**p-value 0.01; ***p-value 0.005, α = 0.05, Chi square test). No statistical differences between DENV and CHKV transmission are shown by asterisk (*p-value 0.25, α = 0.05, Chi square test).

For CHIKV, 69.8% of females (N = 146 fed females) were infected after feeding on blood with 1.7 × 10^7^ PFU/ml. Dissemination of CHIKV began day 5 (40.9%) and reached a peak of 57.8% (N = 102) in infected mosquitoes. Detection in saliva started in females on 10 dpi with a total percentage of 6.2% of individuals with disseminated infections transmitted. The extrinsic incubation period, calculated as the mean day of transmission detection, was 16.5 days for CHIKV, as compared to 21 days for DENV (Table 1).

The proportion of CHIKV-infected mosquitoes was significantly higher than DENV infection (p-value = 0.005, α = 0.05). Likewise, the proportion of mosquitoes with disseminated virus was significantly higher for CHIKV than DENV (p-value 0.01, α = 0.05). However, there was not a significant difference in the proportions of mosquitoes transmitting the two viruses (p-value = 0.25, α = 0.05).

## Discussion

*Aedes albopictus* has spread from its native range to at least 28 other countries around the globe^1^. Several studies demonstrated that variants of *Ae. albopictus* from the Northern and Southern hemispheres show a distinct dispersal behavior^33^. However, the geographic expansion of *Ae. albopictus* across Brazil after the first detection in 1986 from tropical Asia^2^ to at least 20 of the 27 Brazilian states^19^, is quite different from what occurs in Argentina. The current distribution of this species in Argentina is limited to four locations in the province of Misiones^21–23^. In this study we did not detect the presence of *Ae. albopictus* in other locations located along the National Route 12, which connects Posada to Puerto Iguazú. But our search was limited to used car tires, thus to confidently rule out other breeding sites it would be necessary to sample in other types of containers, for instance, those belonging to the natural habitat of *Aedes albopictus*.

Field samples of *Ae. albopictus* were less abundant than other co-existing mosquito species, in agreement with the results of Schweigmann *et al*.^22^ in a nearby area (Eldorado, Misiones province). Furthermore, we obtained differences in some population parameters as compared with other studied populations of *Ae. albopictus* from America and Asia^34–36^. For instance, although larval and pupal survival was high, we found a low hatch rate of eggs, leading to a negative growth rate. Higher rates of egg hatching were recorded in other populations of *Ae. albopictus* strains as is New Orleans (57%) and Houston (93%)^36^. One explanation for the decreased hatch rates and immature abundance of *Ae. albopictus* populations from Misiones province is *Wolbachia* infection status, wich may induce cytoplasmic incompatibility (CI), due to the bacteria is not fixed in this population^37^.

The studied population was polymorphic for *Wolbachia* infection, containing uninfected individuals, individuals superinfected with both *w*AlbA and *w*AlbB strains, or infected only with *w*AlbB. We isolated the strain from the *w*AlbB infected individuals to characterize it for the first time by full MLST, which is useful for comparative purposes. In describing the diversity of *Wolbachia* in this host species, Zhou *et al*.^38^ found the A and B strains using a wsp-based phylogeny. However, Casiraghi *et al*. and Baldo *et al*.^39,40^ reported the mosaic nature of this gene, which may yield misleading results. Previously, Baldo *et al*.^41^ proved that *w*AlbA belongs to the A supergroup by full MLST and here we also used it for *w*AlbB, leading us to confirm its identity as a member of the B supergroup.

Although we recovered the same *Wolbachia* strains already reported for *Ae. albopictus*, prevalence in the population from Iguazú National Park was 80.8%, while the prevalence reported for most populations worldwide is 100%^42–44^. The latter is generally expected since this bacteria tends to rapidly spread to fixation after it invades a population^45^, yet our result is in agreement with the study of Turelli^46^, who concluded that a CI strain showing no fertility cost to infected females, complete phenotype expression and a transmission rate of 80% will reach a polymorphic equilibrium of 0.72, causing a 20% reduction in population fertility. Experimental crossing assays between superinfected males and *w*AlbB infected females will be conducted in order to measure the levels of penetrance of CI in this population and thus ascertain if the low hatch rate observed in the present study is ascribable to this phenotype induced by *Wolbachia*.

Single *w*AlbB infection in males of Iguazú National Park population can be explained by depletion of *w*AlbA infection during the life cycle^47^, while the occurrence of negative individuals is more difficult to interpret. Bacteria could have been undetected because of low loads or by absence of infection and further investigation using real time PCR would be helpful to obtain more accurate results.

We also studied the vector competence of this mosquito population to determine its potential importance in DENV and CHIKV transmission in the northeast area of Argentina. Our results indicated *Ae. albopictus* from subtropical Argentina are competent but relatively inefficient vectors for both CHIKV and DENV. DENV transmission, in particular, was low and delayed, with extrinsic incubation period (EIP) of 21 days identified in a single transmitting individual. The extended EIP and low transmission rates of DENV in *Ae*. a*lbopictus* from Iguazú National Park indicate that this population is unlikely to be a significant contributor to DENV activity in the region. Despite both a shorter EIP and higher rate of dissemination for CHIKV, transmission was still relatively inefficient. At the population level, there was no significant difference between transmission of CHIKV and DENV looking at all mosquitoes exposed to an infectious blood meal. These results are similar to that found by Vega-Rúa *et al*.^7^ and suggest there is either a significant salivary gland infection or transmission barrier preventing highly efficient transmission by this population of *Ae. albopictus*.

Mousson *et al*.^31^ showed that *Wolbachia* naturally infecting *Ae. albopictus* from La Reunion Island limits DENV-2 dissemination and the infection of salivary glands, since removing *Wolbachia* prevented this inhibition. Our data suggest potential different levels of interference with arboviruses by this endosymbiont. *Aedes aegypti* is considered the main epidemic vector of DENV in the Americas, while *Ae. albopictus* is regarded as a secondary vector. Overall, our results with the Argentina population of *Ae. albopictus* are consistent with this characterization and indicate that, in the absence of both population expansion and viral adaptation, *Ae. albopictus* are likely to remain a minor contributor to arbovirus transmission in the region.

## Methods

### Mosquitoes and environmental data collection

A survey of the *Ae. albopictus* that breeds in containers was performed in order to ascertain their current distribution. The collection sites were located all along the National Route 12, the major roadway that connects Posadas to Puerto Iguazú, both located in the Misiones province, in Subtropical Argentina, in the northern part of the country (Fig. 1). All collections were taken from various artificial containers of standing water in the area using a siphon bottle. For Iguazú National Park, which belongs to Parques Nacionales, Argentina, a NEA 326 permit was obtained. Morphological identification of the specimens (3^rd^ and 4^th^ instar larvae) was performed using dichotomous keys^48^, while 1^st^ and 2^nd^ instars and pupae were reared either to the 4^th^ instar or to adult emergence, respectively. Immature stages were transported to the lab forfurther processing. Daily temperature and humidity were recorded by hour using a HOBO data logger during February in Iguazú National Park, where we were able to collect a sample of *Ae. albopictus*. These data were used to build an average curve of maximum to minimum daily cycle of temperature from this location.

### Life table and demographic parameters

We examined life-history traits of *Ae. albopictus* using the first generation (F1). F1 eggs (N = 500) were hatched and placed in an incubator with a temperature cycle fluctuating between 21 °C and 34 °C and a photoperiod 14:10 (L:D), simulating the conditions recorded in the field. Fifty first instar larvae were separated in 1 L of dechlorinated water in a plastic flat tray (30 × 18 × 6 cm) with finely ground guinea pig food. Instar stage and mortality were recorded daily, as well as the day of pupation. The pupae were removed to plastic containers (8 × 3.5 cm diameter), and provided with water and raisins in preparation for emergence. Following emergence, adult were sexed and put in a cardboard cage (25 × 22 cm diameter) for 3 to 5 days to allow mating before blood feeding. After having fed on a hamster for 45–60 min, the adults were moved to separate plastic containers (8 × 3.5 cm diameter, 1 per container) with filter paper and wet cotton on the bottom for oviposition (OVI 1). Seven days after the first feeding, adult females were placed together in a cardboard cage for a second blood feeding and then were placed once again in individual containers to oviposit. Adult mortality was checked daily and dead adults were stored at −20 °C. Eggs laid by each blood-fed female were counted, transferred to a Petri dish on filter paper with cotton, and sealed using parafilm to maintain humidity for 7–10 days. Eggs were then recounted to verify numbers and condition, and put in a plastic container with 250 ml of dechlorinated water and 10 mg of yeast for hatching. This procedure was carried out twice and the eggs that did not hatch were not examined to verify embryogenesis. The larvae were counted 48 hours later. Survival was expressed as the percentage of individuals that reached the next instar/stage. Larval and pupal mean development time and sex ratio of emerged adults were also measured. Fecundity was determined as the total number of eggs laid per mosquito (total number of engorged females) and fertility as the proportion of eggs hatched over the total number of eggs per population. Daily mortality records were used to calculate survival from the first day at the first instar as a function of age (lx); the number of eggs laid daily was used to calculate the age-specific fecundity (mx), by dividing total number of eggs laid each day (x) by the number of individuals alive at the end of that day. The lx and mx schedules allowed the estimation of demographic parameters such as the intrinsic rate of natural increase (*r*), the net reproductive rate (*Ro*), and the mean generation time (*Tg*)^49^.

### Detection of Wolbachia

We extracted DNA from adult mosquitoes (20 females and 8 males) stored at −20 °C from life table study using the Wizard Genomic Purification Kit (Promega). Multiplex PCR was carried out using the temperature profile of 95 °C for 1 min, 55 °C for 1.5 min and 72 °C for 2 min for 35 cycles and *wsp* primers. Primers used were 328 F and 691 R for *w*AlbA strain and 183 F and 691 R for *w*AlbB strain, as described by Zhou *et al*.^38^. When only one strain was detected, and due to the possible competition between both *w*AlbA and *w*AlbB DNAs in the multiplex PCR, we conducted independent PCRs to detect either *w*AlbA or *w*AlbB in such individuals. Negative results from independent PCRs were repeated twice. The quality of DNA extraction was checked using a primer set that amplifies the mosquito cytochrome oxidase 1 mtDNA locus (COI) under the conditions specified by Rodriguero *et al*.^50^. Any sample that yielded a negative result for COI was excluded from the data set. Samples that were negative for *wsp* primers but positive for COI primers were scored as uninfected. All negative results for *Wolbachia* infection were checked twice with fbpA and coxA primers^40^.

### Sequencing of Wolbachia

Strain *w*AlbB was characterized by means of full MLST through amplification and sequencing of the cytochrome oxidase subunit I (*coxA*), fructose-bisphosphate aldolase (*fbpA*), cell division protein ftsZ (*ftsZ*), aspartyl/glutamyl-tRNA amidotransferase subunit B (*gatB*) and conserved hypothetical protein (*hcpA*) fragments using the primers and conditions described in Baldo *et al*.^40^. MLST profile of *w*AlbA strain can be seen in Baldo *et al*.^40^. In addition, we provided the *wsp* sequence, in order to further characterize this strain on the basis of the amino acid motifs of the four hypervariable regions (HVRs) of this sequence.

DNA was purified with a DNA Puriprep-GP (Inbio Highway). DNA fragments were sequenced using a sequencer ABI3730 XL (Macrogen Inc., Korea). Chromatograms were edited using BIOEDIT^51^. Allele number was given to every gene after comparison with the *Wolbachia* MLST database (http://pubmlst.org/wolbachia). Thus, *w*AlbB strain was characterized by the combination of the MLST numbers (allelic profile or ST).

We accomplished a phylogenetic analysis including strains from supergroups A, B, C, D, F and H. The *coxA*, *fbpA*, *ftsZ*, *gatB* and *hcpA* gene sequences were concatenated and aligned using the CLUSTALW algorithm^52^. The terminal units were 52 *Wolbachia* strains retrieved from the *Wolbachia* MLST database and the one obtained in the present work. Our complete data set includes 2079 aligned nucleotide positions.

jModelTest 2^53^ was used to infer the most appropriate model of molecular evolution. The GTR + G model was selected as the best fit model of nucleotide substitution for the *coxA*, *ftsZ*, *gatB* and *hcpA* partitions and the GTR + G + I was the best fit for the *fbpA* partition.

Bayesian phylogenetic analysis of the concatenated MLST sequences was applied through the ‘metropolis-coupled Markov chain Monte Carlo’ (MC3) algorithm implemented in MrBayes v. 3.2.6^54^ using a partitioned algorithm. Two independent analyses were run with a random starting tree over 2,500.000 generations with a sample frequency of 500. The tree space was explored using four chains: one cold and three incrementally heated chains, with temperature (*T*) set to 0.20. The first 500 trees were discarded as burn-in. We assessed stationarity of the cold Markov chain for all MrBayes analyses in TRACER^55^, in addition to the standard deviation of the split frequencies. All posterior samples of a run prior to the burn-inpoint were discarded. Remaining trees were taken into account to obtain a 50% majority-rule consensus tree and mean branch length estimates. The frequency of all bipartitions was estimated to assess the support of each node^56^.

As the root for the overall tree of the *Wolbachia* genus is still undetermined^39,57^, we did not include any outgroup. However, for analyzing horizontal transfer, it is necessary to examine sister group relationships. Thus, we arbitrarily rooted the tree with supergroup H.

### Vector competence assays

#### Peroral infection

These studies were conducted in the BSL-3 insectary at the Arbovirus Laboratory, Wadsworth Center, New York State Department of Health, Albany, New York, USA using F1 eggs from obtained from the Iguazú National Park population. Vector competence assays were conducted using DENV2 strain 306 from Nicaragua (GenBank no. SAMN011003699) and CHIK strain 91077 (GenBank no. EF451145). Blood meals containing virus were prepared using freshly harvested virus following 4 (CHIKV) or 6 (DENV) days of growth on *Ae. albopictus* mosquito cells (C6/36, ATCC). Specifically, 8.5 mL of defibrinated bovine blood and 0.5 mL of 50.0% (w/v) sucrose were used for blood meal preparation, infected cells were scraped and the media with cell suspension was mixed 1:1 with the bloodmeal.

Five to seven day old female adult mosquitoes were allowed to feed on the infected blood meal in sausage casing for 1 hour at room temperature following the protocol of Ebel *et al*.^58^. Blood suspensions were frozen at −80 °C for subsequent plaque assay to determine the virus titer at the time of mosquito feeding. Fully engorged females were separated and held in 0.5 L cartons in incubators with temperature cycles fluctuating between 21 °C and 34 °C and a 12:12 (light:dark) photoperiod, and provided a 10% sucrose solution ad lib on cotton wicks until mosquitoes were harvested. At 5, 10, 14 and 21 days post-feeding females were anesthetized with Triethylamine (Sigma, MO), and bodies, legs, and salivary secretions were obtained from each individual mosquito and processed as previously described to determine infection, dissemination, and transmission rates, respectively^58^.

The number of mosquitoes with virus in legs (dissemination) and salivary secretion (transmission) was calculated as a proportion of the number of mosquitoes that were infected. Rates were compared between groups using Chi square (χ^2^) or Fisher’s exact tests, as appropriate according to the sample sizes. All analyses were performed using R.

## Ethics Statement

The research has been conducted according to Argentine laws following the procedures and protocols approved by Ethics Committee for Research on Laboratory Animals, Farm and Obtained from Nature of National Council of Scientific and Technical Research (CONICET) (Resolution 1047, section 2, annex II), and subsequently by National Agency for the Promotion of Science and Technology of Argentina (ANPCYT) (PICT 2015-0665). For collecting in Iguazú National Park Permit NEA 326 issued by Administración de Parques Nacionales, Argentina (GuíaTránsito 002978). All vector competence studies were conducted in a BSL-3 insectary according to the Guidelines established in the “Arthropod Containment Guidelines” as published (Arthropod containment guidelines. A project of the American Committee of Medical Entomology and American Society of Tropical Medicine and Hygiene. Vector Borne Zoonotic Dis. 2003 Summer; 3(2):61–98). The experimental protocol and facilities were approved by CDC and Wadsworth Institutional Biosafety Committee.
